# Case Report: Globus Pallidus Internus (GPi) Deep Brain Stimulation Induced Keyboard Typing Dysfunction

**DOI:** 10.3389/fnhum.2020.583441

**Published:** 2020-11-09

**Authors:** Joshua K. Wong, Melissa J. Armstrong, Leonardo Almeida, Aparna Wagle Shukla, Addie Patterson, Michael S. Okun, Irene A. Malaty

**Affiliations:** Department of Neurology, McKnight Brain Institute, Norman Fixel Institute for Neurological Diseases, University of Florida, Gainesville, FL, United States

**Keywords:** deep brain stimulation, dystonia, dystypia, connectomics, stimulation induced side effect

## Abstract

**Introduction:**Typing on a keyboard requires complex collaboration between visuospatial/procedural memory, language, and motor function. The impaired ability to type, independent of motor deficits, apraxia, or aphasia has been coined “dystypia.”

**Case Presentation**: A 68-year-old woman with a history of blepharospasm, oromandibular, and segmental dystonia underwent bilateral pallidal deep brain stimulation (DBS) because of a waning response to botulinum toxin therapy. Following DBS, she discovered she no longer “remembered” how to type fluidly and had to “hunt and peck” for letters on the keyboard. This issue persisted at a 2-year follow-up. The patient underwent serial typing tests with the DBS ON vs. OFF. Post-operative lead reconstruction was performed using Lead-DBS. The volume of tissue activation (VTA) modeling was combined with whole-brain tractography.

**Results**: Typing improved when the device was switched to the DBS OFF state. Cortical mapping revealed strong modulation of the right angular gyrus, left calcarine fissure, and left cuneus. There was also activation of bilateral supplemental motor areas and superior parietal gyri.

**Discussion**: Shared lesion topography analysis of dystypia cases in the literature has suggested the involvement of the superior longitudinal fasciculus (SLF). The SLF involves the superior parietal lobe, angular gyrus, supramarginal gyrus, and arcuate fasciculus. Our patient’s connectivity pattern suggested SLF involvement. The improvement in OFF state typing and her imaging together suggested that the dystypia in her case was a stimulation-induced side effect.

**Conclusion**: Dystypia is a rare side effect of Globus Pallidus Internus (GPi) DBS therapy and may be associated with SLF involvement.

## Introduction

Typing on a keyboard is a complex coordinated function that requires network coordination between visuospatial procedural memory, language, and motor function (Ryu et al., 2012; Cook et al., 2013). The impaired ability to type, when manifested independently of motor deficits, apraxia, or aphasia, has been coined “dystypia” (Otsuki et al., 2002). Dystypia can be disruptive and impact activities of daily living especially given the heavy use of technology. In light of recent reports of swimming dysfunction following deep brain stimulation (DBS), we report a case of dystypia (keyboard typing dysfunction) as a potential side effect of DBS therapy (Waldvogel et al., 2020).

## Case Presentation

A 68-year-old woman with a history of blepharospasm, oromandibular and segmental dystonia underwent bilateral pallidal DBS. Before DBS she had a waning response to botulinum toxin therapy. In the acute stage of her recovery from the DBS surgery, she reported “forgetting how to type on the computer.” At baseline, she was proficient in typing as she did not have dystonia symptoms in the arm. Her previous occupation was administrative office work which involved copious typing. Within weeks following DBS implantation, she reported she no longer “remembered” how to type fluidly and had to “hunt and peck” for letters on the keyboard. This issue persisted into the 2-years of follow-up we have for her post-DBS. She did not have issues with any other motor tasks.

The clinicians performed the following testing to sort out the dysfunction and the relationship to DBS activation or inactivation (three conditions): (1) DBS ON; (2) 10 min following the DBS OFF condition; and (3) 20 min following the DBS OFF condition. Informed consent for testing and videotaping was obtained from the patient. Visuospatial testing (e.g., Trail Making B test, cube copy, clock drawing) and the neurological exam were performed in the DBS ON state and it was confirmed that there was no evidence of visuospatial neglect or aphasia. She performed three different 1-min online typing tests which were provided by the National Computer Science Academy (NCSA, Whitesboro, TX, USA). The tests can be viewed in the Supplementary Video 1. The three segments show DBS ON, 10-min washout, and 20-min washout. The testing duration was limited by the return of the patient’s pre-operative dystonia symptoms.

A post-operative DBS lead location analysis was performed using the Lead-DBS advanced image processing pipeline (Horn and Kühn, 2015; Horn et al., 2019)^1^. The volume of tissue activation (VTA) was modeled and estimated based on the patient’s most recent DBS programming parameters [Left Globus Pallidus Internus (GPi): 1 − C + 3.5 V, 150 μs, 120 Hz; Right GPi: 9 − C + 2.5 V, 150 μs, 120 Hz]. Whole-brain deterministic tractography was computed in DSI Studio using a diffusion MRI population-averaged template from the Human Connectome Project (HCP-1021; Yeh et al., 2013, 2018). The VTA was used as a seed for fiber tracking and resulting activated fiber tracks were calculated.

## Results

Video testing of the patient’s typing was performed at 2 years and 7 months post-implantation. The DBS programming settings at the time of testing were unchanged for the previous 2 years. This setting was reported to provide maximal benefit for her dystonia symptoms. Based on the latest settings, she reported complete or near-complete resolution of her dystonia symptoms except for her mild dystonic tremor in the right arm.

As the washout time increased (DBS OFF), she reported increased confidence in her typing abilities and less dependence on looking down at the keyboard. Her typing speed also increased from 0.6 words per minute (WPM) at baseline to 2.6 WPM and 3.8 WPM after turning the DBS OFF for 10 and 20 min, respectively. She felt typing improved OFF DBS but not back to her pre-DBS baseline.

**Figure 1:**
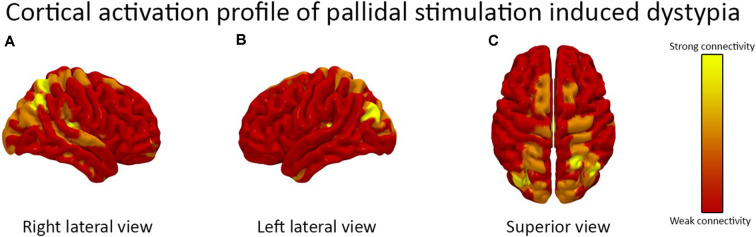
The cortical activation pattern of bilateral globus pallidus internus (GPi) deep brain stimulation (DBS) is demonstrated. The **(A)** right lateral view **(B)** left lateral view and **(C)** superior view are shown. The activation pattern is based on the patient’s DBS device programming parameters. There was a strong activation of the right angular gyrus, left calcarine fissure, and left cuneus and moderate activation of bilateral supplemental motor areas and the superior parietal gyri.

The fiber tract activation pattern from the patient’s DBS lead location analysis is shown in Figure 1. Cortical parcellation mapping revealed a strong modulation of the right angular gyrus, left calcarine fissure, and left cuneus. There was the activation of bilateral supplemental motor areas and the superior parietal gyri.

## Discussion

Several case reports have described “dystypia” in the absence of motor weakness, apraxia, and aphasia (Otsuki et al., 2002; Ryu et al., 2012; Cook et al., 2013; Thomas and Mestre, 2017; Sharma et al., 2019). Typically, dystypia has been reported in the setting of acute stroke. One case report described a patient with dystypia following bilateral subthalamic nucleus (STN) DBS (Lee et al., 2016). Post-operative imaging revealed prominent vasogenic edema in the frontal lobe surrounding the left STN DBS lead. In contrast to this case, Lee et al. (2016) attributed the dystypia to frontoparietal network dysfunction secondary to peri-electrode edema rather than a stimulation-induced side effect of STN DBS. The authors observed a marked improvement in dystypia following resolution of the peri-electrode edema. A recent literature review investigating the shared lesion topography of dystypia cases proposed involvement of the superior longitudinal fasciculus (SLF; Sharma et al., 2019). The SLF has been characterized to involve the superior frontal lobe, dorsal prefrontal region, superior parietal lobe, the angular gyrus, the supramarginal gyrus, and the arcuate fasciculus (Makris et al., 2005). A comparison to our patient’s connectivity pattern also revealed the involvement of the SLF as a possible explanation for her dystypia.

We acknowledge several limitations of this study. First, we recognize our case is an N-of-1 observation and that these results should be interpreted with caution. However, the findings in our study highlight that there may be underrecognized stimulation-induced DBS related side effects. As these impairments appear to be task-specific and patient-specific, we appreciate the need for a larger, more robust exploration into stimulation-induced side effects. Second, due to limitations in the MRI acquisition, our connectomic analysis utilized population-averaged template data rather than patient-specific data. Future studies should incorporate both approaches to see if there are any differences in connectivity results. Finally, we acknowledge the need in future cases for a comprehensive neuropsychological testing battery to further characterize dystypia.

Our findings suggest that dystypia is a rare stimulation-induced side effect of GPi DBS therapy and may be related to SLF involvement. This phenomenon appears different than GPi induced parkinsonism, however, comparative studies should be performed to observe if parkinsonism involves a common network pathway (Blahak et al., 2011; Schrader et al., 2011). Although the fundamental phenomenology between dystypia and parkinsonism is different, the examination of the active contacts, lead location, and fiber bundles stimulated by GPi DBS may reveal overlapping connectivity profiles or modulation of interconnected nodes along the same network. Recent reports of DBS-induced swimming dysfunction may share common underpinnings and this approach may be useful to better understand this phenomenon (Waldvogel et al., 2020). Finally, since the majority of centers have yet to formally assess for dystypia post-DBS, the prevalence of this DBS side effect remains unknown.

## Data Availability Statement

The datasets presented in this article are not readily available. Requests to access the datasets should be directed to joshua.wong@neurology.ufl.edu.

## Ethics Statement

Ethical review and approval was not required for the study on human participants in accordance with the local legislation and institutional requirements. The patients/participants provided their written informed consent to participate in this study.

## Author Contributions

JW designed the study, acquired and analyzed the data, and drafted the manuscript. MA, LA, AW, AP, and MO revised the manuscript. IM designed the study and revised the manuscript. All authors contributed to the article and approved the submitted version.

## Conflict of Interest

The authors declare that the research was conducted in the absence of any commercial or financial relationships that could be construed as a potential conflict of interest. The reviewer CZ declared a past co-authorship with several of the authors LA, MO to the handling Editor.

## Footnotes

^1^https://www.lead-dbs.org/

## Supplementary Material

SUPPLEMENTARY VIDEO 1 Pallidal deep brain stimulation (DBS)-induced dystypia. The patient in the video begins by taking a typing test with the DBS ON. Her hands are positioned off-center and she frequently looks down at the keyboard for visual confirmation of each keystroke before pressing down. She primarily uses her index finger to type and must pause to double-check her progress on the screen. After turning the DBS OFF for 10 min, her fingers are positioned over the “home row” keys and she can type more fluidly. She does not constantly look down at the keyboard. After turning the DBS OFF for 20 min, she incorporates all fingers into her typing and she spends most of the time viewing the screen.
